# Subtle emotional mechanisms in postnatal depression: Shame, guilt, and the fragmented maternal identity

**DOI:** 10.1192/j.eurpsy.2026.11390

**Published:** 2026-07-31

**Authors:** C. V. Valeriu

**Affiliations:** Department of Mental Health, Medical Psychology and Psychotherapy, Nicolae Testemițanu State University of Medicine and Pharmacy, Chisinau, Moldova, Republic of

## Abstract

**Introduction:**

Postnatal depression (PND) is frequently a crisis of maternal identity. Shame (“I am a bad mother”) and guilt (“I failed my baby/partner”) intensify isolation and perfectionistic self-attack, undermining engagement with care.

**Objectives:**

To clarify how shame and guilt shape the phenomenology and severity of PND and to identify psychotherapeutic strategies that integrate fragmented maternal self-representations and improve bonding.

**Methods:**

Following PRISMA 2020, we conducted a systematic narrative review of MEDLINE, Embase, PsycINFO, Web of Science, Cochrane Library, and Scopus - covering all records available up to August 2025, without language limits. Eligible studies enrolled women ≤12 months postpartum with diagnosed or screen-positive PND (EPDS/PHQ-9), tested early psychotherapy (≤6 months postpartum; CBT, IPT, attachment-focused, tele-enabled) against usual care or wait-list, and reported depressive outcomes plus at least one measure of shame/guilt or bonding (e.g., TOSCA, GASP, ISS). Designs included RCTs, quasi-experiments, cohorts, and mixed-methods. Risk of bias used RoB 2/ROBINS-I/CASP; certainty followed GRADE. Heterogeneity precluded meta-analysis; synthesis was narrative.

**Results:**

Across studies, early psychotherapy reduced depressive symptoms and attenuated shame/guilt, with parallel improvements in maternal–infant bonding and partner communication. Effects were generally small-to-moderate yet clinically meaningful on EPDS. Mechanistically, therapy shifted appraisals from global defectiveness to specific, repairable behaviors, strengthened self-compassion and regulation, and enabled graded disclosures that disconfirmed anticipated rejection. Attachment-infused CBT/IPT enhanced durability; tele-enabled care matched in-person outcomes within screening-to-referral pathways. Limitations included heterogeneous measures and attrition; overall bias was low–moderate.

**Conclusions:**

Targeting shame and guilt as active mechanisms—paired with validated early screening and rapid access to evidence-based psychotherapy—reduces symptoms, integrates maternal identity, and strengthens bonding. Future trials should harmonize measures, extend follow-up, and test brief, group, and digital formats for scalable perinatal care.

**Disclosure of Interest:**

None Declared

